# A signature based on anoikis-related genes for the evaluation of prognosis, immunoinfiltration, mutation, and therapeutic response in ovarian cancer

**DOI:** 10.3389/fendo.2023.1193622

**Published:** 2023-06-13

**Authors:** Yiqi Duan, Xiao Xu

**Affiliations:** ^1^ Department of Pharmacy, First Affiliated Hospital of Zhengzhou University, Zhengzhou, Henan, China; ^2^ Department of Obstetrics and Gynecology, Zhongshan Hospital, Fudan University, Shanghai, China

**Keywords:** anoikis-related gene pairs, ovarian cancer, prognostic signature, immunoinfiltration, therapeutic response

## Abstract

**Background:**

Ovarian cancer (OC) is a highly lethal and aggressive gynecologic cancer, with an overall survival rate that has shown little improvement over the decades. Robust models are urgently needed to distinguish high-risk cases and predict reliable treatment options for OC. Although anoikis-related genes (ARGs) have been reported to contribute to tumor growth and metastasis, their prognostic value in OC remains unknown. The purpose of this study was to construct an ARG pair (ARGP)-based prognostic signature for patients with OC and elucidate the potential mechanism underlying the involvement of ARGs in OC progression.

**Methods:**

The RNA-sequencing and clinical information data of OC patients were obtained from The Center Genome Atlas (TCGA) and Gene Expression Omnibus (GEO) databases. A novel algorithm based on pairwise comparison was utilized to select ARGPs, followed by the Least Absolute Shrinkage and Selection Operator Cox analysis to construct a prognostic signature. The predictive ability of the model was validated using an external dataset, a receiver operating characteristic curve, and stratification analysis. The immune microenvironment and the proportion of immune cells were analyzed in high- and low-risk OC cases using seven algorithms. Gene set enrichment analysis and weighted gene co-expression network analysis were performed to investigate the potential mechanisms of ARGs in OC occurrence and prognosis.

**Results:**

The 19-ARGP signature was identified as an important prognostic predictor for 1-, 2-, and 3-year overall survival of patients with OC. Gene function enrichment analysis showed that the high-risk group was characterized by the infiltration of immunosuppressive cells and the enrichment of adherence-related signaling pathway, suggesting that ARGs were involved in OC progression by mediating immune escape and tumor metastasis.

**Conclusion:**

We constructed a reliable ARGP prognostic signature of OC, and our findings suggested that ARGs exerted a vital interplay in OC immune microenvironment and therapeutic response. These insights provided valuable information regarding the molecular mechanisms underlying this disease and potential targeted therapies.

## 1 Introduction

Ovarian cancer (OC) is the most lethal of all female gynecologic cancers worldwide (1). OC is often referred to as the “silent killer” owing to atypical symptoms, such as abdominal bloating and ascites, and a lack of definitive screening tools, leading to its diagnosis in the advanced stages (2). High-grade serous OC accounts for up to 70% of all cases, with many patients experiencing relapse and drug resistance after initial treatment (3). The conventional management of OC includes optimal debulking surgery and platinum-based chemotherapy. With advances in neoadjuvant chemotherapy, cytoreductive surgery, and molecular targeted therapy, the clinical outcome of OC patients has improved significantly (4). However, overall survival (OS) in OC has improved minimally over the last decades. Therefore, developing more effective prognostic models and exploring innovative therapeutic targets for predicting and enhancing outcomes are urgent in OC.

Anoikis is a form of programmed cell death that occurs due to detachment from the extracellular matrix. This regulatory process is essential for inhibiting tumor invasion and metastasis in cancer (5). A critical step in tumor metastasis is the development of anoikis resistance (6). Numerous studies have shown that anoikis plays a vital role in OC. *In vitro* experiments suggest that *LRRC15*, *NOTCH3*, and *PCMT1* promote OC cells’ migration and adhesion by hindering the anoikis-induced cell death (7–9). *CPT1A* and *CBX2* are overexpressed in high-grade serous OC and act as drivers of anoikis resistance and tumor dissemination (10, 11). In contrast, angiopoietin-like protein 2, an inflammatory factor, inhibits peritoneal metastasis of OC cells by suppressing anoikis resistance (12). Recently, some prognostic models based on anoikis-related genes (ARGs) using RNA-sequencing data have been developed for predicting the clinical outcomes of several cancers, including hepatocellular carcinoma (13), cutaneous melanoma (14), gastric cancer (15), lung adenocarcinoma (16), glioblastoma (17), and colorectal cancer (18). However, few studies have systematically evaluated the prognostic value of ARGs in OC. Therefore, a more in-depth and detailed analysis is urgent to explore the prognostic value of ARGs in OC.

In the present study, we adopted a novel algorithm that employed pairwise comparison to identify valid anoikis-related gene pairs (ARGPs) and generated a prognostic signature of 19 ARGPs. The 19-ARGP signature was shown to be reliable and valid in predicting the OS of OC patients and was identified as an independent predictive factor in OC. Furthermore, we revealed potential relationships between ARGs and the immune microenvironment, as well as their potential value in therapy.

## 2 Materials and methods

### 2.1 Data collection and preprocessing

We obtained the HTSeq-FPKM raw data and relevant clinical information data of OC cases from The Center Genome Atlas (TCGA) database ((https://portal.gdc.cancer.gov/) as the training set. After excluding patients without complete survival data, a total of 365 OC patients with complete follow-up data and their follow-up time greater than 30 days were included. In the process of further verification, we adopted the same inclusion criteria and downloaded the GSE9891 (*n* = 229) dataset of OC cases from the Gene Expression Omnibus (GEO) database (https://www.ncbi.nlm.nih.gov/geo/). All datasets were freely available and publicly accessible from the TCGA database or the GEO database. This study was strictly complied with the data extraction policies of the database, and the ethics committee approval was not required to conduct the current study. The raw data were preprocessed to carry out batch normalization to offset the deviations in datasets using “*Combat*” function in R’s “*sva*” package (19). ARGs were obtained from the GeneCards database (https://www.genecards.org/Search/Keyword?queryString=anoikis). A total of 434 ARGs were acquired from the GeneCards database (relevance score>0.4).

### 2.2 Identification of ARGPs in patients with OC

Univariate Cox regression analysis of datasets was performed using R’s “*survival*” package for identifying prognostic ARGs, and those ARGs with *p*< 0.05 were selected (*n* = 52). We pairwise compared the relative expression levels of prognostic ARGs in each sample and scored each ARG (20). ARGs were paired to constitute ARG pairs (ARGPs), including ARG1 and ARG2. If the expression level of ARG1 was lower than that of ARG2 in a particular ARGP, the score of this pair was recorded as 1; otherwise, the score was recorded as 0. This approach based on pairwise comparison could remove the technical heterogeneity and does not require additional standardization because the score entirely depends on gene expression levels in samples. ARGPs generally received the same score (0 or 1) in more than 80% of the samples, possibly attributable to (1) the dependence on platform priority measurements, which could lead to bias and may not be reproducible across platforms, and (2) the biological priority transcription, which could not provide discriminatory information about patients’ survival. Therefore, we deleted these ARGPs with constant value in each dataset and obtained 431 ARGPs. Finally, univariate Cox regression analysis of ARGP was performed using R’s “*survival*” package to identify prognostic AGRPs (*n* = 50).

### 2.3 Construction, validation, and assessment of an ARGP prognostic signature

The data of OC cases from the TCGA database were utilized as the training cohort. The Least Absolute Shrinkage and Selection Operator (LASSO) penalized Cox regression analysis was performed to further screen and remove collinearity for 50 ARGPs. Finally, a risk score model with *β* (*Coef*) value multiplied by the ARGP score was established. Risk score = (*β*1*ARGP1+*β*2*ARGP2+*β*3*ARGP3+…+*β*n*ARGPn), where x*i* and *β* (*Coef*) are the relative expression level and coefficient of ARGP (21, 22), respectively. The risk score formula was as follows:


$$\text{Risk score}=\sum_{i=1}^{n}x_{i}*Coef$$

Finally, a total of 19 ARGPs were identified for prognostic signature building. A total of 366 patients were divided into high- and low-risk groups with the cutoff thresholds of median risk score. Kaplan–Meier survival analysis and log-rank test were performed to generate survival curves for OS and compare the differences between high- and low-risk cases using R’s “*survival*” and “*survminer*“ packages, respectively. The 1-, 2-, and 3-year receiver operating characteristic (ROC) curves and their area under the ROC curves (AUC) were evaluated. The prognostic accuracy of the risk score and other clinical information, such as age, were assessed using univariate and multivariate Cox regression analyses. The predictive power was assessed using a nomogram and time-dependent ROC curves. The discriminative ability of the nomogram was evaluated using calibration curves.

### 2.4 Weighted gene co-expression network analysis and functional enrichment analysis

Weighted gene co-expression network analysis (WGCNA) could cluster genes with similar expression patterns and find the association between modules and specific phonotypes. The signature has well quantified the risk in patients with OC. To elucidate the reason for the significant heterogeneity between the high- and low-risk groups, we constructed a weighted gene co-expression network using R’s “*WGCNA*” package with approximately scale-free characteristics (23). An adjacency matrix was determined by these genes’ expression correlation. The gene modules were produced using the method of topological overlap measure (TOM) (24). Co-expression genes modules and clustering graphs were performed using the dynamic tree cutting algorithm (25). Afterwards, the modules with related genes were merged into new modules. Gene significance (GS) and module significance (MS; the mean value of all GS values) were calculated to measure the correlation between genes and modules. We further identified the gene module (blue module) that was significantly co-expressed in the high-risk group and performed functional enrichment analysis for this module. Furthermore, we used the dataset of C2–C8 from the Molecular Signature Database (MSigDB) database for gene set enrichment analysis (GSEA) to comprehensively analyze the activity differences in the pathway between high- and low-risk groups, and verify the results of the previous functional enrichment analysis (26).

### 2.5 Immune infiltration analysis

We analyzed the difference in immune cell infiltration between high- and low-risk groups, and the association between signature and immuno-infiltration scores using the following R packages: “*MCPcounter*” (27), “*CIBERSORT*” (28), “*xCell*” (29), “*TIMER*” (30), “*EPIC*” (31), “*CIBERSORT-ABS*” (32), and “*QUANTISEQ*” (33). Stromal scores in malignant tissue were estimated using the “Estimation of STromal and Immune cells in Malignant Tumors using Expression data” (*ESTIMATE*) algorithm (34). The Wilcox test was utilized to compare the differences of stromal scores between high- and low-risk groups. Fisher’s exact test was utilized to evaluate the correlation between risk scores and stromal scores.

### 2.6 Mutation profile analysis

We obtained the somatic mutation data of patients with OC from the TCGA database (https://portal.gdc.cancer.gov/). Waterfall graphs were utilized to show the differences in mutation between high- and low-risk groups. R’s “*maftools*” package was utilized to analyze, annotate, and visualize the mutation profile of this signature. The “*plotmafSummary*” function was applied to show the mutation landscape in high- and low-risk groups.

### 2.7 Prediction of drug therapy response

Drug IC_50_ values were downloaded from the Genomics of Drug Sensitivity in Cancer (GDSC) database (https://www.cancerrxgene.org/). We calculated the differences of drug IC_50_ values between the high- and low-risk cases using R’s “*pRRophetic*” package to guide clinical medication (35).

### 2.8 Statistical analysis

R software was utilized to perform all statistical analyses in this study, and statistical significance was set at *p*< 0.05.

## 3 Results

### 3.1 Identification of prognostic ARGs and ARGPs

**Figure 1:**
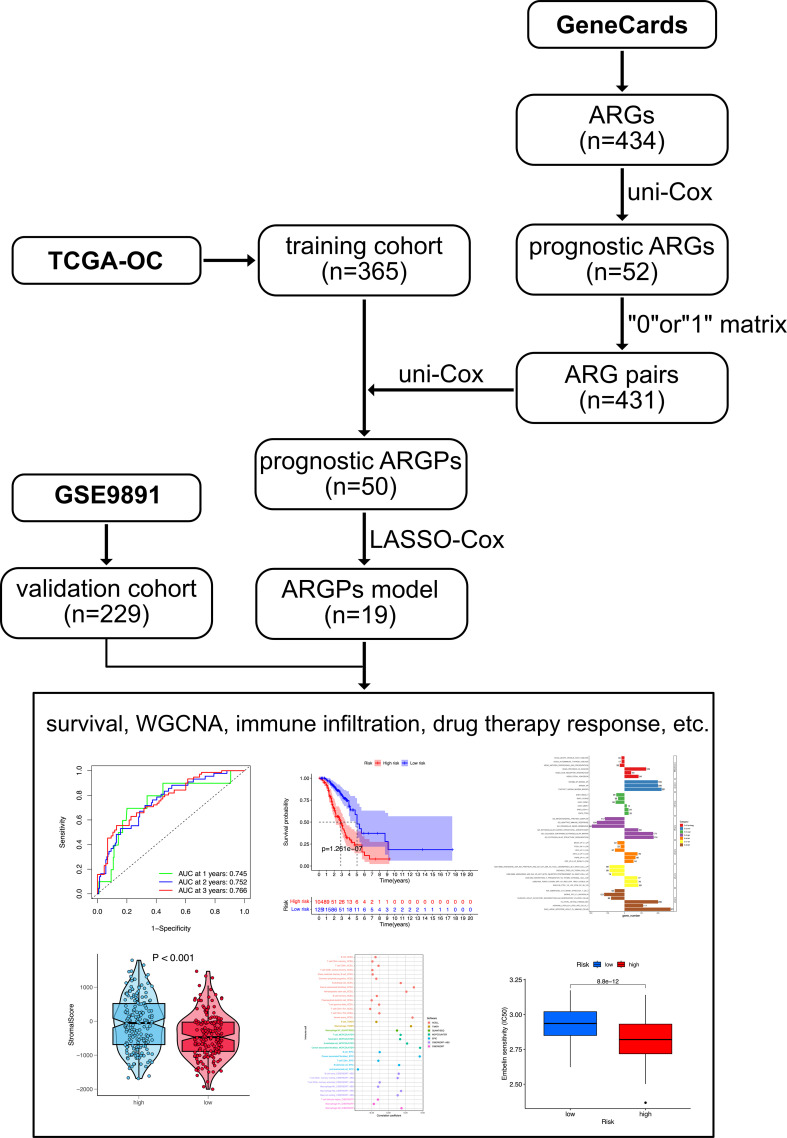
The flowchart of the data analysis.

**Figure 2:**
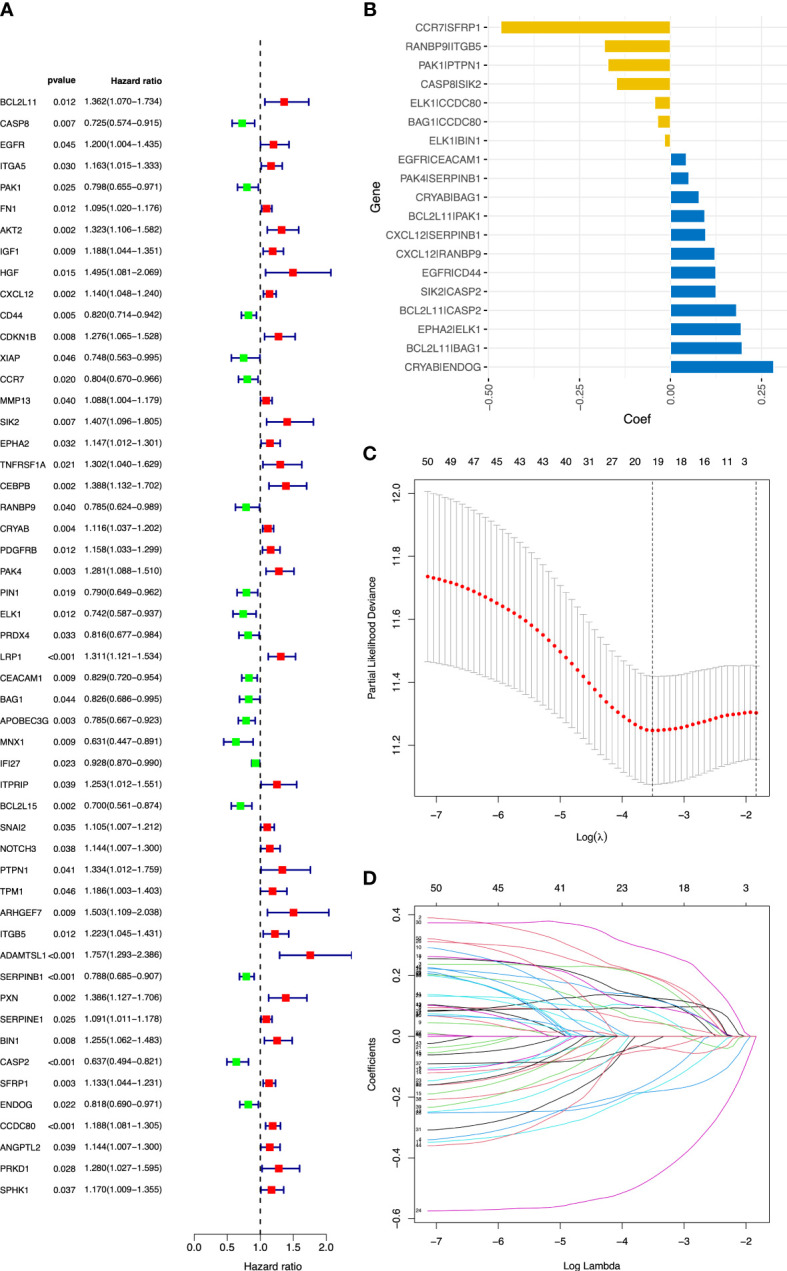
Construction of the ARGP signature in OC patients. **(A)** The forest plot of 52 candidate prognostic ARGs using univariable Cox regression analysis. ARGs with a hazard ratio > 1 are considered as prognostic risk factors. **(B)** Coefficient of the 19 ARGPs. **(C)** Parameter selection by the LASSO algorithm. **(D)** Trend graph of LASSO coefficients. ARGPs, anoikis-related gene pairs; OC, ovarian cancer; TCGA, The Cancer Genome Atlas; LASSO, Least Absolute Shrinkage and Selection Operator.

The flowchart of this study is shown in **Figure 1**. OC samples from the TCGA database (*n* = 365, excluding the patients without complete survival data) were analyzed as the training cohort, and the dataset GSE9891 (*n* = 229) from the GEO database was analyzed as part of the validation cohort. A total of 434 ARGs (relevance score > 0.4) were obtained from the GeneCards database. Univariable Cox regression analysis was performed to identify prognostic ARGs (*n* = 52, *p* value <0.05) and prognostic ARGPs (*n* = 50) using R’s “*survival*” package (**Figure 2A** **;** **Supplementary Table 1**).

### 3.2 Construction and validation of an ARGP prognostic signature

**Figure 3:**
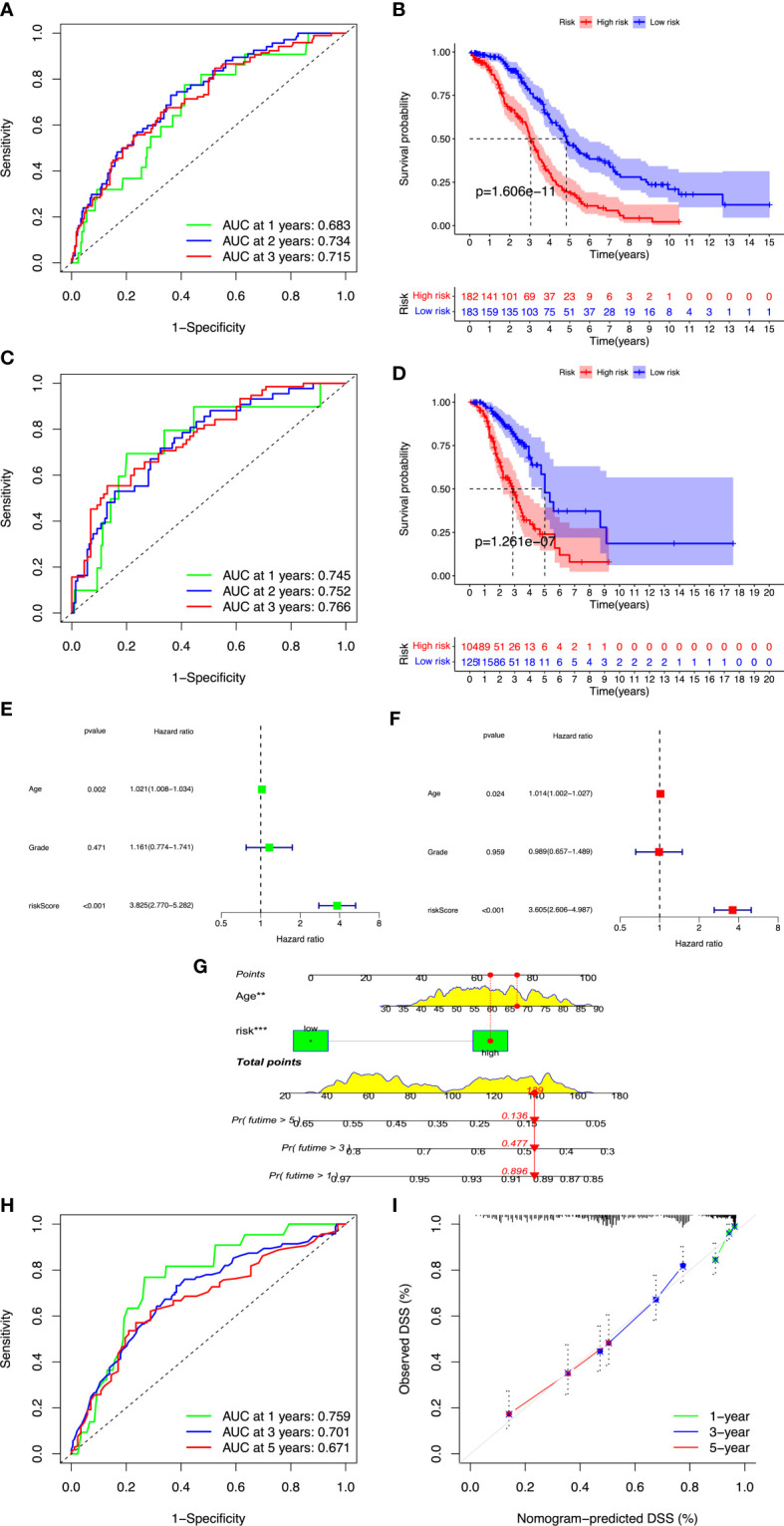
Validation of the ARGP model. OC cases were classified into high- and low-risk groups by median risk score. **(A, B)** Time-dependent ROC curves for predicting OS at 1, 2, and 3 years in the training and validation cohort. **(C, D)** Kaplan–Meier curves of OS in the training and validation cohort. **(E)** The forest plot of univariate Cox regression analysis for the prognosis of OC patients. **(F)** The forest plot of multivariate Cox regression analysis for the prognosis of OC patients. **(G)** The nomogram was constructed based on age and risk scores. **(H)** ROC curves of the nomogram predicting OS at 1, 2, and 3 years. **(I)** Calibration plots of OS at 1, 2, and 3 years. OS, overall survival; ROC, receiver operating characteristic. **p<0.01; ***p<0.001.

After screening and removing collinearity via the LASSO penalized Cox regression analysis, a risk score model with the *β* (*Coef)* value multiplied by the ARGP score was established. A total of 19 ARGPs had significant associations with OS (*p*< 0.001) in OC and were identified for prognostic signature construction (**Table 1** **;** **Figures 2B–D**). A total of 366 patients were divided into high- and low-risk groups with the cutoff thresholds of median risk score. We predicted the OS in the training and validation datasets. One-, 2-, and 3-year time-dependent ROC curves were plotted for the training cohort dataset. The AUCs at 1, 2, and 3 years were 0.683, 0.734, and 0.715 in the training cohort, respectively (**Figure 3A**). The Kaplan–Meier curves showed that OS in the high-risk group was worse than that in the low-risk group (*p* = 1.6062 × 10^−11^) (**Figure 3B**). An independent validation set, GSE9891 (*n* = 229), was utilized for confirming the consistency of the prognostic value of the novel ARGP signature. The same approaches were performed to calculate risk scores and divide them into high- and low-risk groups with the cutoff thresholds of median risk score. In this validation cohort, the AUCs at 1, 2, and 3 years were 0.745, 0.752, and 0.766, respectively (**Figure 3C**). The patients in the high-risk group had worse OS than the patients in the low-risk group (**Figure 3D**). Next, univariable and multivariable Cox regression analyses were performed for evaluating the effects of age, tumor grade, and ARGP risk score on the time-independent ROC curve of the risk score in the training cohort. Univariable Cox regression analysis exhibited a significant association of the 19-ARGPs prognostic signature with OS (hazard ratio [HR] =3.852, 95% confidence interval [CI]: 2.770–5.282; *p*< 0.001) (**Figure 3E**). Multivariable Cox regression analysis showed that the 19-ARGPs prognostic signature was a prognostic factor in patients with OC, independently of age and tumor grade (HR = 3.605, 95% CI: 2.606–4.987; *p*< 0.001) (**Figure 3F**). Then, we established a nomogram including age and risk scores to evaluate OC prognosis (**Figure 3G**). ROC curves (**Figure 3H**) and calibration curves (**Figure 3I**) of the model proved that the nomogram had a great predictive performance.

**Table 1:** Nineteen ARGPs used for the construction of the prognostic risk model.

ARGPs	HR	HR.95L	HR.95H	*p*-value	Coef
BCL2L11|PAK1	1.47333803	1.17937649	1.84056996	0.00064251	0.09480493
BCL2L11|BAG1	1.58561102	1.25861827	1.99755746	9.16E-05	0.19745688
BCL2L11|CASP2	1.63155084	1.26741604	2.10030334	0.00014523	0.18205579
CASP8|SIK2	0.63387534	0.49000651	0.8199849	0.00051851	-0.148909
EGFR|CD44	1.53410392	1.20319668	1.95601839	0.00055606	0.12497917
EGFR|CEACAM1	1.72837356	1.32675224	2.25156972	5.00E-05	0.04406857
PAK1|PTPN1	0.67154817	0.5361234	0.84118125	0.00053018	-0.1724271
CXCL12|RANBP9	1.70957506	1.35174158	2.16213435	7.63E-06	0.12245401
CXCL12|SERPINB1	1.650946	1.30842036	2.08313993	2.38E-05	0.09693771
CCR7|SFRP1	0.59902015	0.44330899	0.80942445	0.00084817	-0.4663205
SIK2|CASP2	1.66572202	1.28949977	2.15171024	9.36E-05	0.12576852
EPHA2|ELK1	1.46364272	1.17054749	1.83012652	0.00083428	0.19446517
RANBP9|ITGB5	0.6841881	0.5460297	0.85730383	0.00097445	-0.1820706
CRYAB|BAG1	1.84019723	1.36868529	2.47414498	5.39E-05	0.07910198
CRYAB|ENDOG	1.76440589	1.32520848	2.34916104	0.00010111	0.28384764
PAK4|SERPINB1	1.52888397	1.21930998	1.91705656	0.00023539	0.05102467
ELK1|BIN1	0.67644187	0.53649342	0.85289695	0.00094839	-0.0170117
ELK1|CCDC80	0.55782112	0.43482044	0.71561586	4.38E-06	-0.0436929
BAG1|CCDC80	0.57643064	0.45701078	0.72705568	3.30E-06	-0.0357301

### 3.3 WGCNA and functional enrichment analysis

**Figure 4:**
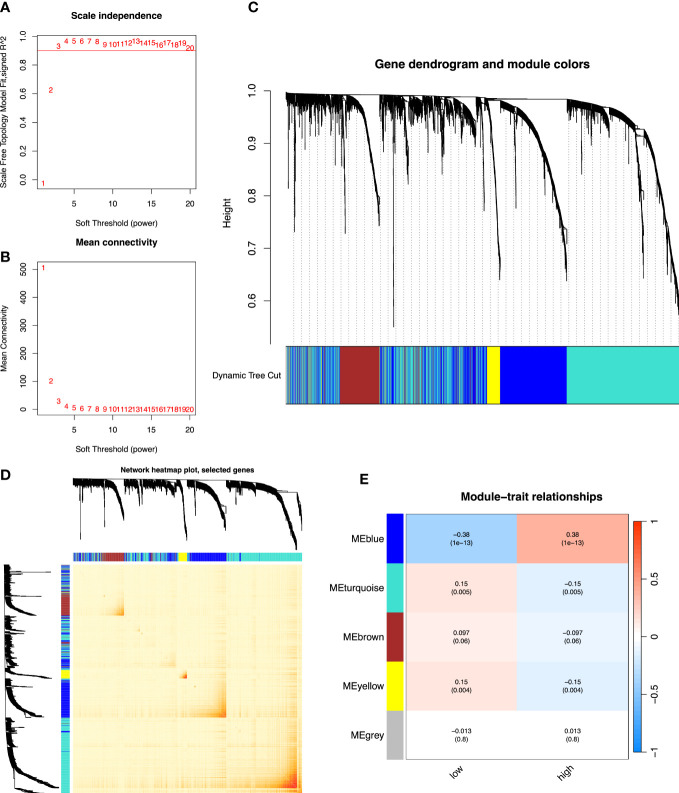
The co-expression network established using WGCNA. **(A)** The *x*-axis suggests the soft-thresholding power value. The *y*-axis represents the scale-free fit index. **(B)** The *y*-axis represents the mean connectivity. **(C)** Clustering dendrogram of genes on the basis of a dissimilarity measure. Different colors reflect gene modules. **(D)** The heatmap shows the visualization of WGCNA network. **(E)** Module–trait relationships. WGCNA, weighted gene co-expression network analysis.

**Figure 5:**
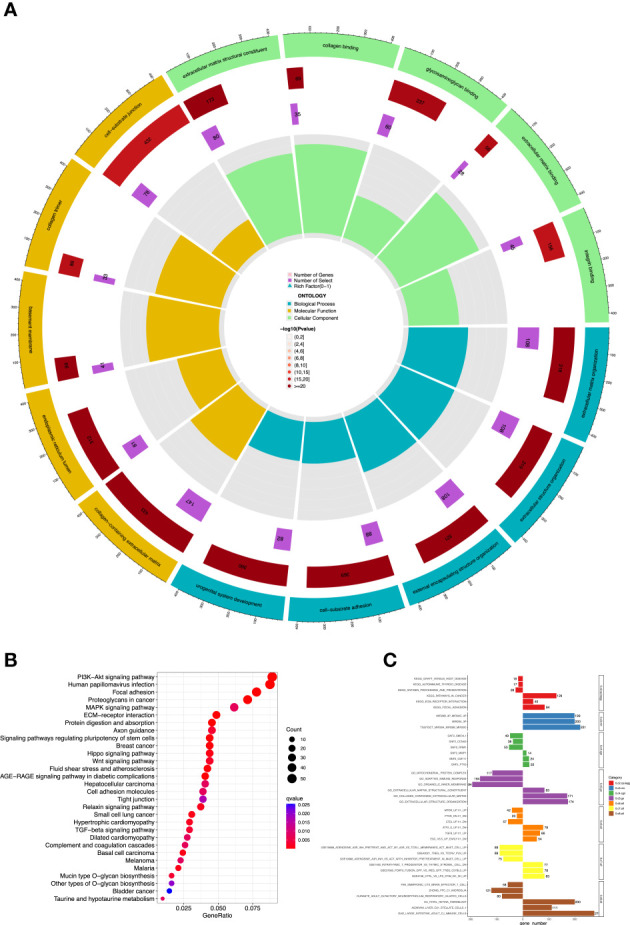
Functional enrichment analysis of 19-ARGPs. **(A)** Significant enriched GO analysis. **(B)** Significantly enriched KEGG pathways. **(C)** The GSEA reveals these most significant enrichment pathways. GO, Gene Ontology; KEGG, Kyoto Encyclopedia of Genes and Genomes; GSEA, gene set enrichment analysis.

R’s “*pickSoftThreshold*” package was utilized to calculate the soft thresholding power *β* (set at 3), in which the scale independence reached 0.9 (**Figure 4A**) and had a relatively high-average connectivity (**Figure 4B**). We constructed a weighted gene co-expression network using R’s “*WGCNA*” package with approximately scale-free characteristics. Co-expression gene modules and clustering graphs were performed using the dynamic tree cutting algorithm. Five gene co-expression modules were finally constructed (**Figure 4C**). We used the method of TOM and mapped the relationships between the identified gene modules (**Figure 4D**). The light color represented a low overlap, and the dark red color represented a high overlap. These results revealed that the gene expression level was relatively independent between modules. Afterwards, the correlated modules were merged into new modules. The results showed that five modules could be clustered into blue, turquoise, brown, yellow, and gray modules, respectively (**Figure 4E**). We further calculated the GS and MS, and identified the gene module blue, which was mostly significantly correlated with high-risk cases (*p*< 0.001). Then, we performed GO and KEGG analyses in the blue module. The GO analysis showed that (**Figure 5A**), regarding the biological process, the genes were mainly enriched in extracellular structure and matrix organization, as well as external encapsulating organization. As for the cellular component, the genes were mainly enriched in extracellular matrix structural constituent, collagen binding, and extracellular matrix binding. As for molecular function, the genes were mainly enriched in basement membrane and collagen-containing extracellular matrix. The KEGG analysis showed that the blue module pathways include the PI3K-Akt signaling pathway, human papillomavirus infection, focal adhesion, and ECM–receptor interaction (**Figure 5B**). Finally, the dataset of C2–C8 from the MSigDB database was used for GSEA to comprehensively analyze the differences in signaling pathway activation between high- and low-risk groups, and confirm the results of the previous functional enrichment analysis. The GSEA revealed that the genes were mainly enriched in pathways in cancer, ECM–receptor interaction, focal adhesion, extracellular structure organization, immune response, and collagen-containing extracellular matrix (**Figure 5C**). Consequently, the GSEA results were almost in agreement with the conclusions obtained from GO and KEGG analyses.

### 3.4 Assessment of immune infiltration

**Figure 6:**
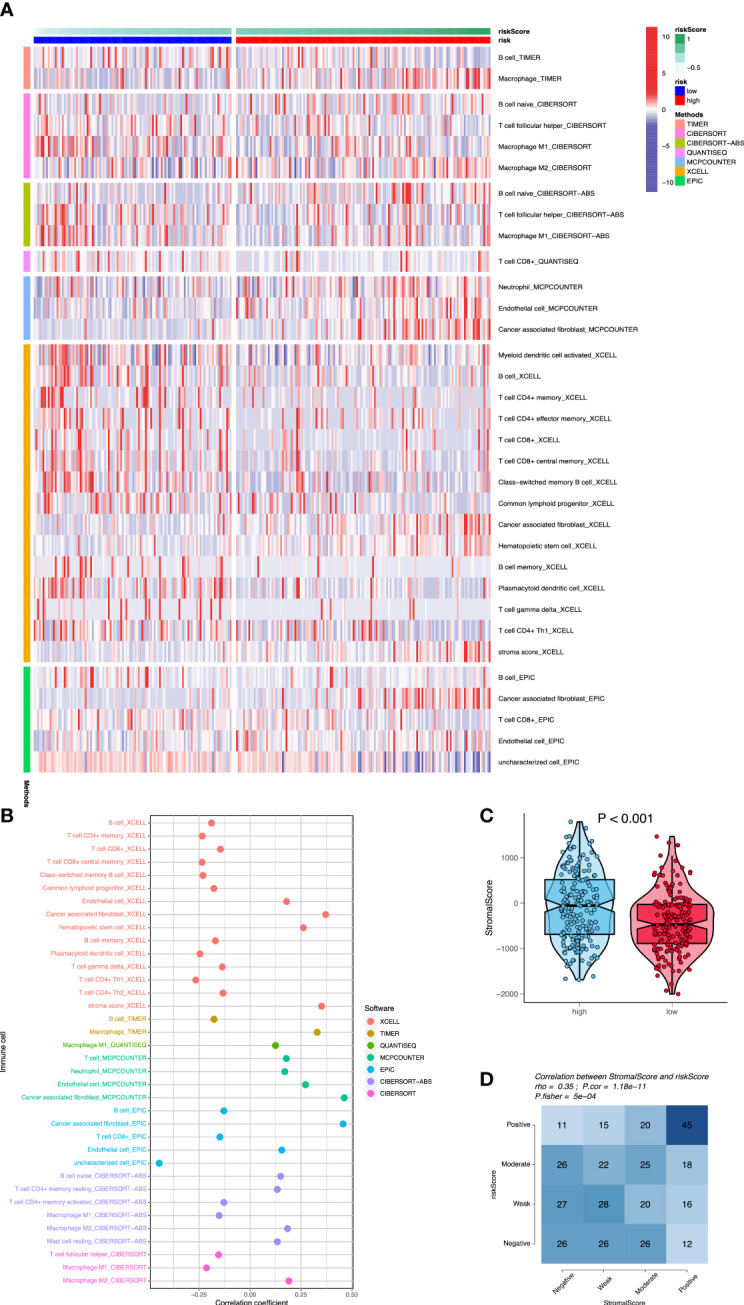
Assessment of immune cell infiltration between the two risk groups. **(A)** The heatmap shows that differences of immune cell infiltration between high- and low-risk groups using seven algorithms. **(B)** The correlation analysis of immune cells using bubble chart. **(C)** The differences of stromal scores between high- and low-risk cases. **(D)** The correlation analysis between stromal scores and risk scores.

A major direction is to investigate the infiltration of immune cells in the immune microenvironment of OC. We compared the differences in immune cell infiltration between high- and low-risk groups, and the association between signature and immuno-infiltration scores using the following R packages: “*MCPcounter*”, “*CIBERSORT*”, “*xCell*”, “*TIMER*”, “*EPIC*”, “*CIBERSORT-ABS*”, and “*QUANTISEQ*”. Stromal scores were calculated by the “ESTIMATE” algorithm. Fourteen immune cell types showed significantly different proportions between the high- and low-risk groups (*p*< 0.05) (**Figure 6A**). In particular, M2 macrophages, neutrophils, endothelial cells, and cancer-associated fibroblasts (CAFs) had elevated infiltration levels in high-risk cases compared with those in low-risk cases, whereas M1 macrophages, CD4+ memory T cells, CD4+ Th1 T cells, CD8+ T cells, CD8+ central memory T cells, class-switched memory B cells, common lymphoid progenitors, hematopoietic stem cells, and plasmacytoid dendritic cells had decreased proportions (all *p*< 0.01). Among them, the infiltration of M2 macrophages, neutrophils, endothelial cells, and CAFs was positively correlated with the risk score of OC patients (all *p*< 0.001). The infiltration of M1 macrophages, CD4+ memory T cells, CD4+ Th1 T cells, CD8+ central memory T cells, class-switched memory B cells, common lymphoid progenitors, hematopoietic stem cells, and plasmacytoid dendritic cells was negatively correlated with the risk score of OC patients (all *p*< 0.001) (**Figure 6B**). Furthermore, the Wilcox test showed that stromal scores in high-risk cases were higher than those in low-risk cases (*p*< 0.001) (**Figure 6C**). Fisher’s exact test showed that stromal scores were positively correlated with risk scores (*p*< 0.001) (**Figure 6D**). In high-risk patients with OC, high stromal scores reflected the presence of mesenchymal cells that had been reported previously (36). These results suggested that OC patients in the high-risk group could be more prone to immune escape due to the existence of immunosuppressive microenvironment, resulting in tumor recurrence and metastasis.

### 3.5 Identification of mutation profiles

**Figure 7:**
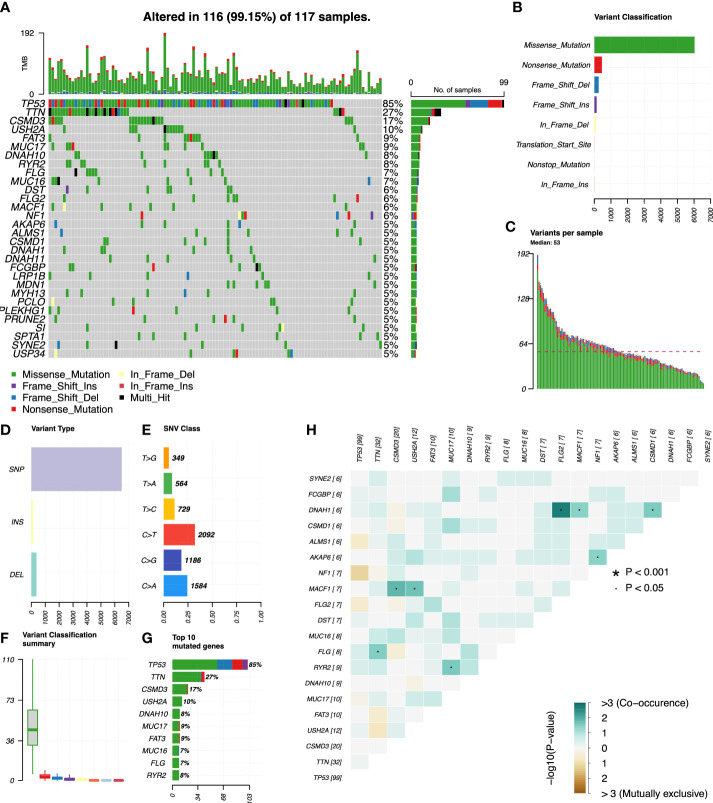
Mutation profiles in the high-risk group of OC patients. **(A)** Waterfall plot reflects the top 30 frequent mutation genes. **(B)** Eight common variant classifications in all samples. **(C)** Variations per sample. **(D)** Three variant types in all samples. **(E)** Six classes of SNV. **(F)** Summary of variant classification. **(G)** Top 10 mutated genes. **(H)** The correlation analysis of mutated genes. *p<0.001; ·p<0.05.

**Figure 8:**
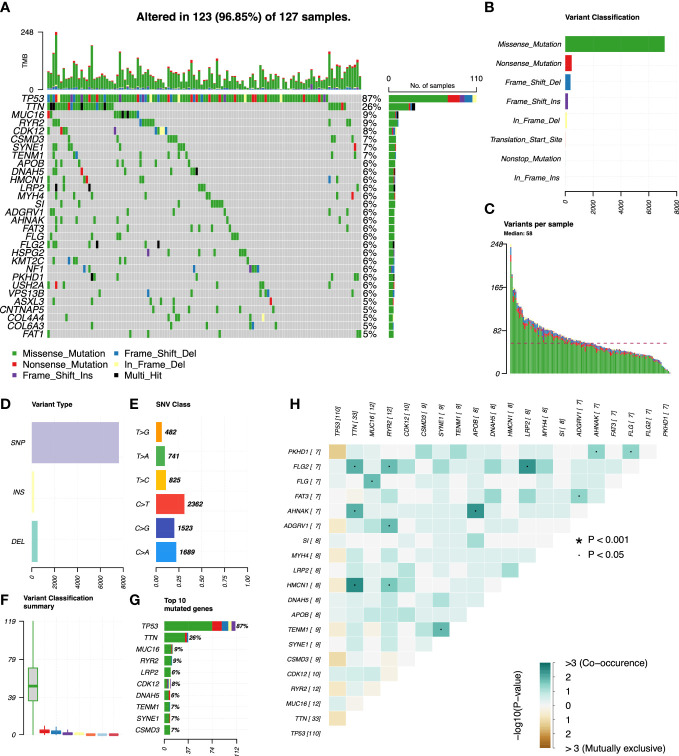
Mutation profiles in the low-risk group of OC patients. **(A)** Waterfall plot reflects the top 30 frequent mutation genes. **(B)** Eight common variant classifications in all samples. **(C)** Variations per sample. **(D)** Three variant types in all samples. **(E)** Six classes of SNV. **(F)** Summary of variant classification. **(G)** Top 10 mutated genes. **(H)** The correlation analysis of mutated genes. *p<0.001; ·p<0.05.

We used R’s “*maftools*” package to analyze, annotate, and visualize the mutation profiles of this signature. The “*plotmafSummary*” function was performed to show the somatic mutation profiles in high- and low-risk groups of OC patients. In the high-risk group, a waterfall plot revealed that approximately 116 of 117 (99.15%) samples showed somatic mutations (**Figure 7A**). In the low-risk group, approximately 123 of 127 (96.85%) samples showed somatic mutations (**Figure 8A**). Among them, the top 10 mutated genes in the high- and low-risk groups are shown in **Figures 7G**, **8G**, respectively. TP53 was the most frequent mutated gene in high-risk (85%) and low-risk (87%) cases, followed by TTN (27%) in high-risk (27%) and low-risk (26%) cases. Other mutated genes were different in these two groups. In the high-risk group, CSMD3 (17%) and USH2A (10%) were common mutated genes. In contrast, MUC16 (9%) and RYR2 (9%) were common mutated genes in the low-risk group. The overall levels of tumor mutation burden (TMB) in high-risk cases (192 mutations) were lower than those in low-risk cases (248 mutations). Next, gene mutations were classified into eight types. Missense mutation was the most frequent variation type in both groups, followed by nonsense mutation, deletion, and insertion (**Figures 7B**, **8B**). Single-nucleotide polymorphism mutated more frequently than insertion and deletion, and C>T was the most frequent single-nucleotide variant class (**Figures 7D, E**, **8D, E**). Furthermore, variant number in each sample was calculated in high-risk (median number: 53) and low-risk groups (median number: 58) (**Figures 7C**, **8C**). Boxplot revealed the variant classification with different colors **(** **Figures 7F**, **8F**). The differences of co-occurrence and mutually exclusive expression in mutation profiles between the two groups are shown in **Figures 7H**, **8H**.

### 3.6 Prediction of drug therapy response

**Figure 9:**
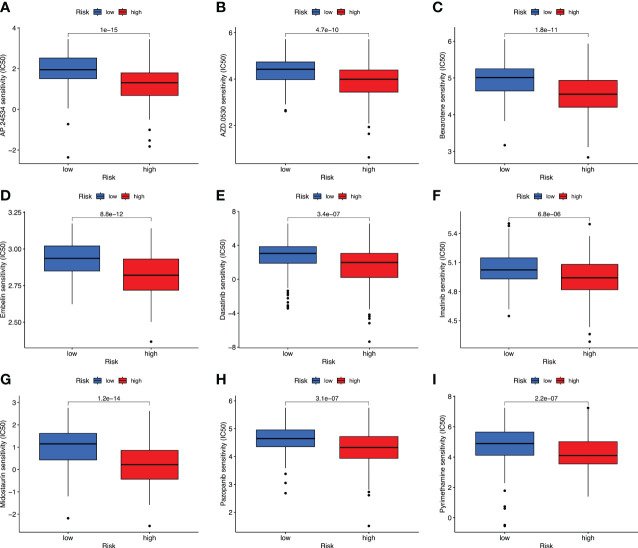
Prediction of drug therapy response. Boxplot of IC_50_ values for drugs including AP.24534 **(A)**, AZD.0530 **(B)**, Bexarotene **(C)**, Embelin **(D)**, Dasatinib **(E)**, Imatinib **(F)**, Midostaurin **(G)**, Pazopanib **(H)**, and Pyrimethamine **(I)** in high- and low-risk groups.

**Figure 10:**
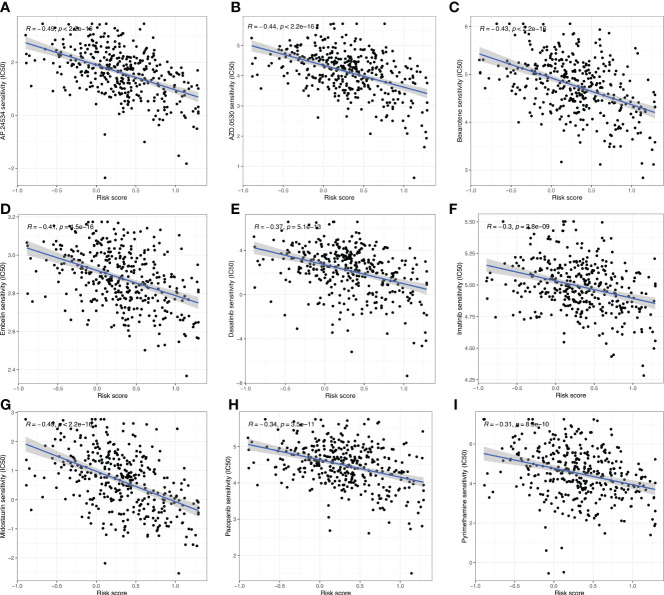
The correlation analysis between the IC_50_ value of drugs and the risk score. These drugs include AP.24534 **(A)**, AZD.0530 **(B)**, Bexarotene **(C)**, Embelin **(D)**, Dasatinib **(E)**, Imatinib **(F)**, Midostaurin **(G)**, Pazopanib **(H)**, and Pyrimethamine **(I)**.

Targeted therapy plays a critical role in many aspects of OC management, and the relationship between risk score and targeted therapeutic response was analyzed. We downloaded the half-maximal inhibitory concentration (IC_50_) values of drugs from the GDSC website and calculated the differences in IC_50_ values between the high- and low-risk groups using R’s “*pRRophetic*” package. High-risk cases were more sensitive to AP.24534 (ponatinib), AZD.0530 (saracatinib), Bexarotene, Embelin, Dasatinib, Imatinib, Midostaurin, Pazopanib, and Pyrimethamine (*p*< 0.001) (**Figures 9A–I**, **10A–I**). These results confirmed the validity of the ARGP signature in predicting targeted therapy response. It is a valuable guidance for the medication of OC patients.

## 4 Discussion

OC is known for its insidious onset and poor prognosis. Even with decades of advancements in OC treatment, the 5-year OS rate has not shown significant improvement (37). Anoikis, a type of programmed cell death, plays an important role in tumor progression and metastasis. ARGs have been confirmed to be involved in the occurrence and development of OC. However, the prognostic values of ARGs in OC have not been elucidated. Considering the important role of ARGs in OC, we constructed an ARGPs-based model to predict the prognosis of OC cases. In this study, we identified 52 prognostic ARGs and created 431 ARGPs. Among them, 50 ARGPs with prognostic values were found (**Supplementary Table 1**). We found that BCL2L11|PAK1, BCL2L11|BAG1, BCL2L11|CASP2, EGFR|CD44, EGFR|CEACAM1, ITGA5|CD44, ITGA5|PIN1, FN1|IFI27, CXCL12|CD4, CXCL12|XIAP, CXCL12|RANBP9, SIK2|CASP2, CRYAB|BAG1, CRYAB|ENDOG, PDGFRB|PIN1, PDGFRB|SERPINB1, PAK4|SERPINB1, LRP1|BAG1, LRP1|APOBEC3G, LRP1|CASP2, LRP1|ENDOG, and ITGB5|SERPINB1 were prognostic risk factors (HR > 1). Other gene pairs were prognostic protection factors in OC. In particular, *BCL2L11*, *CD44*, and *PAK1* were reported as the independent prognosis markers of OC (38–40). Expect for the three genes, other genes were identified for the first time as prognostic factors in OC. Genes *EGFR, XIAP, PIN1*, and *PDGFRB* play important roles in the occurrence and progression of OC and may act as the therapeutic targets in OC (41–43). The expression levels of *ITGA5, CRYAB, IFI27*, and *CEACAM6* genes were significantly increased in high-grade OC, which promoted OC metastasis (44–46). *IFI27* may be related to platinum resistance of OC (47). *Endonuclease G (ENDOG)* and *BAG1* promote OC cell proliferation (48) by regulating EGF signaling pathway and enhancing anoikis resistance (49). Furthermore, the roles of *LRP1, RANBP9*, *SERPINB1*, and *CASP2* genes in OC have not been reported in previous studies, which need to be explored.

We pairwise compared the expression levels of prognostic ARGs in each sample and innovatively calculated the ARGP score. A total of 19 ARGPs were used for the construction of the prognostic model. Among them, CASP8|SIK2, PAK1|PTPN1, CCR7|SFRP1, RANBP9|ITGB5, ELK1|BIN1, ELK1|CCDC80, and BAG1|CCDC80 are prognostic protection factors (HR< 1). The downregulated expression of caspase-8 in OC may be linked to high aggressiveness with chronic inflammation and immune resistance (50). As for chemotherapy, *SIK2* decreases sensitivity of OC cells to paclitaxel and promotes migration of OC cells (51, 52). *PTPN1* is overexpressed in high-grade serous OC, and may act as a marker of better chemotherapy response (53). Furthermore, *SIK2* and *CCR7* both participate in epithelial–mesenchymal transition and promote OC cell migration and invasion (54), whereas *SFRP1* and *BIN1* both inhibit the epithelial OC through inhibiting Wnt/β-catenin signaling (55, 56). Steroid hormones also regulate ARGs and are involved in OC occurrence. For example, follicle-stimulating hormone can stimulate phosphorylated Elk-1 in OC cells (57). High expression levels of *APOBEC3G* and *ITGB5* may be correlated with improved outcomes in high-grade OC patients (58, 59). These ARGs were found for the first time as prognostic protection factors in OC. More in-depth clinical research is necessary to explore their prognostic protection function.

In this study, we found that OC patients in the high-risk group had worse OS than the low-risk group. External validation dataset and internal hierarchical verification revealed the great prediction power of this signature, providing a reliable and effective tool for clinicians to evaluate the survival of OC patients. Furthermore, we explored the potential function of a 19-ARGP signature in OC using immune cell infiltration analysis and WGCNA combined with functional enriched analysis. High proportions of immunosuppressive cells, such as M2 macrophages, and CAFs were found in the high-risk cases. M1 macrophages, CD4+ memory T cells, CD4+ Th1 T cells, CD8+ T cells, CD8+ central memory T cells, class-switched memory B cells, common lymphoid progenitors, hematopoietic stem cells, and plasmacytoid dendritic cells had high proportions in low-risk cases. These results suggest that immunosuppressive microenvironment probably promotes OC progression and metastasis by mediating immune scape. CAFs could recruit ITGA5^high^ ascitic tumor cells to form metastatic units, which further sustain ascitic OC cells’ ITGA5 expression by EGF secretion (44). CAFs also induce epithelial–mesenchymal transition in OC by CXCL12/CXCR4 and PAK1/β-catenin signaling pathways (60). The regulation of ARGs in the immune microenvironment needs further investigation.

At present, many clinical trials focus on targeted therapeutics. We further explored the drug response of patients with OC in two groups. Except for the nine drugs mentioned above, other compounds were also identified, for which the high-risk group showed higher sensitivity than the low-risk group (**Supplementary Figures 1**, **2**). Somatic mutations are considered as the key of immunotherapy in tumors (61). In this study, the proportion of somatic mutations in high-risk cases (99.15%) were higher than that in low-risk cases (96.85%). High proportions of somatic mutations will lead to an increase in neoantigen production, thus improving the immune therapy response. These results require clinical trials to further elucidate and verify.

There are some limitations in our study. This is a retrospective study with data from the TCGA and GEO databases, lacking some clinical information, such as therapeutic and prognostic data. Our results require further investigation about patients with OC in clinical settings. At the same time, a portion of the prognostic genes related to the anoikis that we have obtained still lack sufficient *in vitro* and *in vivo* experiment verification in OC.

## 5 Conclusion

In conclusion, we established a novel and reliable 19-ARGP prognostic signature for predicting the OS of OC patients and analyzed the association between the immune microenvironment and OC. Furthermore, this study indicated that the risk score was relevant to immune cells’ infiltration and somatic mutation. Finally, the ARGP signature may help identify patients with OC suitable for targeted therapy and act as a promising predictive factor to offer insights into therapeutic strategies.

## Data availability statement

The original contributions presented in the study are included in the article/**Supplementary Material**. Further inquiries can be directed to the corresponding author.

## Author contributions

YD and XX performed the study and analyzed the data. XX wrote the manuscript. YD participated in the study design and helped revised the manuscript. All authors contributed to the article and approved the submitted version.

## Supplementary material

Supplementary Table 1 Identification of 50 prognostic ARGPs using the R’s “survival” package.

Supplementary Figure 1 Differences in drug therapy response between high- and low-risk groups.

Supplementary Figure 2 The correlation analysis between IC50 value of compounds and the risk score.
