# Supplementary figures and images for: A signature based on anoikis-related genes for the evaluation of prognosis, immunoinfiltration, mutation, and therapeutic response in ovarian cancer
Source: Front Endocrinol (Lausanne). 2023 Jun 13;14:1193622. doi: 10.3389/fendo.2023.1193622 (PMC10295154; doi:10.3389/fendo.2023.1193622)

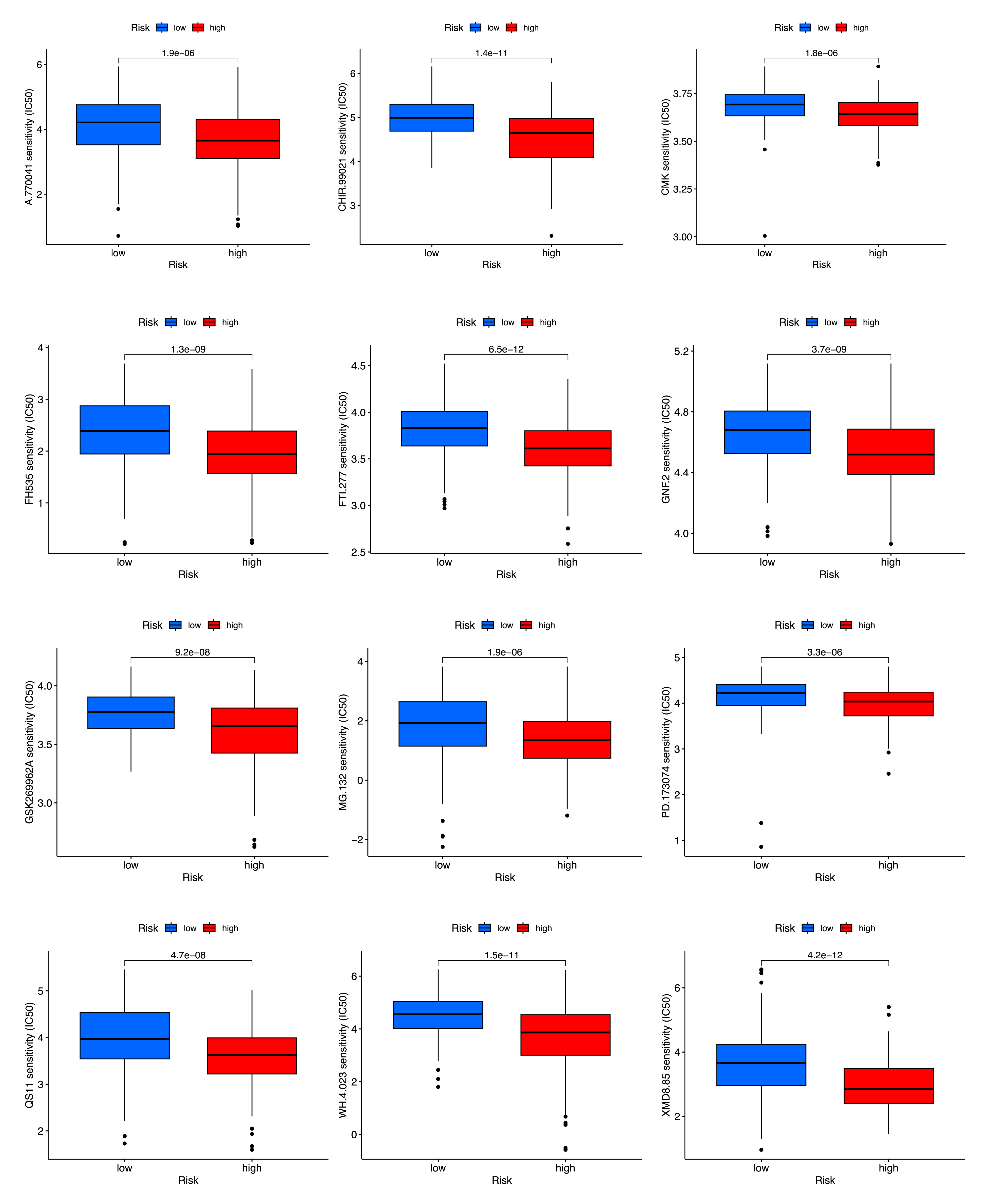

Supplement: Supplementary Figure 1 — Differences in drug therapy response between high- and low-risk groups. [file Image_1.tiff]

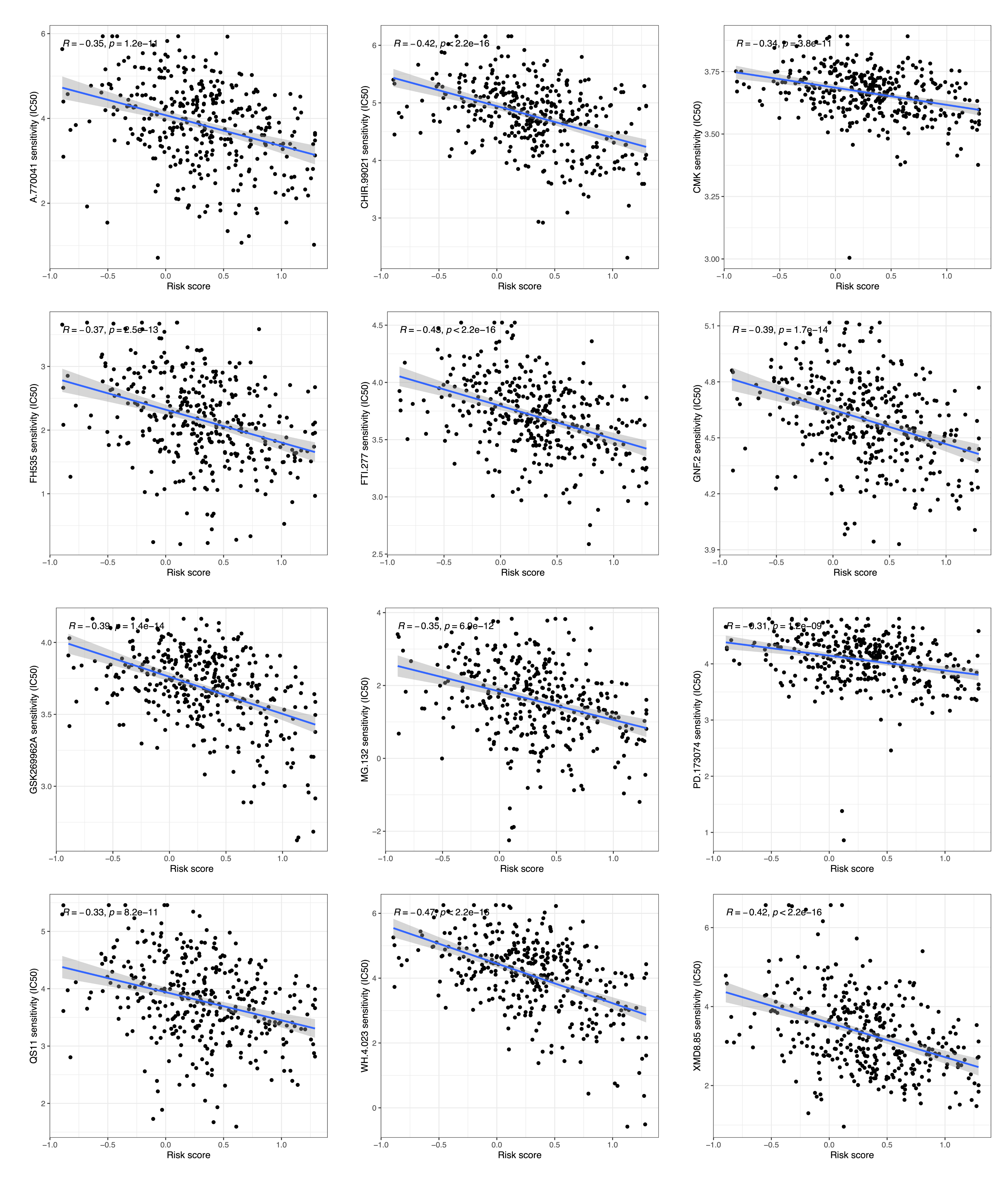

Supplement: Supplementary Figure 2 — The correlation analysis between IC50 value of compounds and the risk score. [file Image_2.tiff]
